# CYP2J2^∗^7 Genotype Predicts Risk of Chemotherapy-Induced Hematologic Toxicity and Reduced Relative Dose Intensity in Ethiopian Breast Cancer Patients

**DOI:** 10.3389/fphar.2019.00481

**Published:** 2019-05-14

**Authors:** Jemal Hussien Ahmed, Eyasu Makonnen, Getnet Yimer, Daniel Seifu, Abebe Bekele, Mathewos Assefa, Abraham Aseffa, Rawleigh Howe, Alan Fotoohi, Moustapha Hassan, Eleni Aklillu

**Affiliations:** ^1^Department of Pharmacology, Addis Ababa University, Addis Ababa, Ethiopia; ^2^Department of Pharmacy, Jimma University, Jimma, Ethiopia; ^3^Division of Clinical Pharmacology, Department of Laboratory of Medicine, Karolinska Institutet Huddinge, Stockholm, Sweden; ^4^Center for Inovative Drug Development and Therapeutic Trials, Addis Ababa University, Addis Ababa, Ethiopia; ^5^Department of Biochemistry, Addis Ababa University, Addis Ababa, Ethiopia; ^6^Department of Surgery, Addis Ababa University, Addis Ababa, Ethiopia; ^7^Department of Oncology, Addis Ababa University, Addis Ababa, Ethiopia; ^8^Armauer Hansen Research Institute, Addis Ababa, Ethiopia; ^9^Clinical Pharmacology Unit, Department of Medicine, Karolinska Institutet, Karolinska University Hospital, Stockholm, Sweden; ^10^Department of Laboratory Medicine, Experimental Cancer Medicine, Clinical Research Centre, Karolinska Institutet, Stockholm, Sweden

**Keywords:** *CYP2J2*, *CYP2C9*, chemotherapy, hematologic toxicity, reduced relative dose intensity, breast cancer, Ethiopia

## Abstract

Chemotherapy-induced hematologic toxicity is the primary reasons of dose reductions and/or delays, low relative dose intensity (RDI), and predicts anticancer response. We investigated the incidence and predictors of chemotherapy-induced hematologic toxicities and reduced RDI in Ethiopian breast cancer patients, and implication of pharmacogenetics variations. Breast cancer patients (*n* = 249) were enrolled prospectively to receive cyclophosphamide based chemotherapy. Hematological toxicity (neutropenia, anemia, and thrombocytopenia) were monitored throughout chemotherapy cycle. The primary and secondary outcomes were incidence of grade 3 or 4 toxicity and reduced RDI, respectively. *CYP2B6^∗^6, CYP3A5^∗^3, CYP2C9 (^∗^2,^∗^3*), *CYP2C19 (^∗^2,^∗^3), CYP2J2^∗^7, POR^∗^28*, and *ABCB1 (rs3842)* genotyping were done. Cox proportional hazard and logistic regression were used to estimate risk predictors of toxicity and reduced RDI, respectively. Majority (73.5%) of the patients were < 45 years of age. The incidence of grade 3 or 4 hematological toxicity was 51.0% (95% CI = 44.54–57.46%). Multivariate Cox proportional hazard regression indicated *CYP2J2^∗^7* genotype [Hazard ratio (HR) = 1.82; 95% CI = 1.14–2.90], pretreatment grade 1 leukopenia (HR = 2.75; 95% CI = 1.47–5.15) or grade 1 or 2 neutropenia (HR = 2.75; 95% CI = 1.73–4.35) as significant predictors of hematologic toxicities. The odds of having hematologic toxicities was lower in *CYP2C9^∗^2 or ^∗^3* carriers (*p* = 0.024). The prevalence of reduced RDI was 56.6% (95% CI = 50.3–62.9%). Higher risk of reduced RDI was associated with *CYP2J2^∗^7* allele [Adjusted odds ratio (AOR) = 2.79; 95% CI = 1.21–6.46], BMI ≤ 18.4 kg/m^2^ (AOR = 5.98; 95% CI = 1.36–26.23), baseline grade 1 leukopenia (AOR = 6.09; 95% CI = 1.24–29.98), and baseline neutropenia (AOR = 3.37; 95% CI = 1.41–8.05). The odds of receiving reduced RDI was lower in patients with *CYP2B6 ^∗^6/^∗^6* genotype (AOR = 0.19; 95% CI = 0.06–0.77). We report high incidence of chemotherapy-induced hematological toxicities causing larger proportion of patients to receive reduced RDI in Ethiopian breast cancer patients. Patients carrying *CYP2J2^∗^7* allele and low baseline blood counts are at a higher risk for chemotherapy-induced hematologic toxicities and receiving reduced RDI, and may require prior support and close follow up during chemotherapy.

## Introduction

Breast cancer has become the most commonly diagnosed cancer in women of Sub-Saharan African countries (Jemal et al., 2012). Though national figure regarding the prevalence of cancer in Ethiopia are non-existent, according to the Addis Ababa cancer registry (AACR), breast cancer accounts for 33% of cancer cases followed by cervical cancer (17%) (AACR, 2014). Chemotherapy plays a crucial role in increasing the cure rate of early breast cancer, provided that the appropriate dose is administered at recommended treatment schedule (Havrilesky et al., 2015). However, cancer chemotherapeutic regimens are known to suppress the hematopoietic system (Kozma et al., 2012), and hematologic toxicities remain the most common reasons for delaying treatment schedule and reducing relative dose intensity (RDI). Chemotherapy induced hematological toxicities are one of the key factors that affect delivery of chemotherapy. The prevalence of chemotherapy induced hematologic toxicities and complications vary between patients depending on type of treatment regimen received (Lyman et al., 2005; Crawford, 2006) and use of supportive care therapies such as recombinant granulocyte colony stimulating factor (G-CSF) which minimize the risk and maintain planned dose of chemotherapy on time (Crawford, 2006).

Various studies show that achieving maximal benefit of anticancer chemotherapy requires the maintenance of dose intensity (Bonadonna et al., 2005; Terada et al., 2009). Dose intensity (DI) is defined as the total amount of drug delivered to a patient per unit time; while RDI is the ratio of the actual dose intensity delivered to the standard dose intensity for a chemotherapy regimen (Citron, 2004; Vavra et al., 2013). Patients who received RDI of 85% or more of the standard dose have a longer relapse free and overall survival, while treatment with below this threshold of RDI is associated with poor survival outcomes (Bonadonna et al., 1995). There is a strong association between RDI and disease-free and overall survival, in both early stage and metastatic cancers especially for lymphoma (Terada et al., 2009) and breast cancer (Sandy and Della-Fiorentina, 2013; Vavra et al., 2013). Thus, RDI can be considered to be a clinical quality indicator and partly a surrogate marker for survival (Wildiers and Reiser, 2011).

Anthracycline-based or taxane-plus-anthracycline-based chemotherapy containing cyclophosphamide regimens are commonly used to treat early stage, locally advanced or metastatic breast cancer in resource limited settings (Anderson et al., 2006). Metabolic pathways of these drugs involve genetically polymorphic drug metabolizing enzymes and transporter proteins. Cyclophosphamide is a prodrug requiring bioactivation by genetically polymorphic cytochrome P450 enzymes that affect its disposition, including *CYP2B6* (Xie et al., 2003), *CYP2C9, CYP2C19* and *CYP3A4*/5 (Roy et al., 1999), and *CYP2J2* (El-Serafi et al., 2015). Several anticancer drugs including cyclophosphamide and doxorubicin are substrates of p-glycoprotein encoded by the polymorphic *ABCB1* gene (Leith et al., 1999). *ABCB1* variant alleles are associated with reduced clearance and significantly increased exposure level of doxorubicin (Lal et al., 2008). Some of the variant alleles are specific or occur at a higher frequencies in black Africans population than whites and Asians (Gebeyehu et al., 2011; Aklillu et al., 2016), and their impact on toxicities associated with treatment of common infectious diseases in sub-Saharan Africa is well characterized (Mugusi et al., 2012, 2018; Mukonzo et al., 2013; Yimer et al., 2014). However their impact on breast cancer associated toxicity remain to be investigated.

Characteristics of breast cancer and its treatment outcomes vary widely across patients and ethnically diverse populations (O’Donnell and Dollan, 2009), partly due to genetic variation (Westbrook, 2013). Breast cancer in black African women is commonly characterized by younger age of onset, as clinically aggressive, with high prevalence of triple negative tumor and higher mortality rates than age-matched Caucasian women (Morris and Mitchell, 2008; Lund et al., 2009). Treatment outcomes and pharmacogenetics of breast cancer chemotherapy are not well investigated in population of Sub-Saharan Africa. In Ethiopia, one retrospective study reported that the 5 years cumulative probabilities of distant metastasis-free survival (MFS) for breast cancer as being 72% (for stages 1 and 2) and 33% (for stage 3) (Kantelhardt et al., 2014). Yet, data on chemotherapy induced hematological toxicities, reduced chemotherapy dose intensities and associated risks from black Africans breast cancer patients including Ethiopians is lacking. Assessment of the incidence and identification of risk factors including pharmacogenetic markers for severe toxicities and reduced dose intensity is essential for personalized care and treatment. Thus, the objective of this study was to assess the incidence and prognostic markers including pharmacogenetics of chemotherapy induced hematologic toxicities and reduced RDI in female breast cancer patients from Ethiopia.

## Materials and Methods

### Study Population and Design

This prospective cohort study was conducted at the radiotherapy center of Tikur Anbessa specialized hospital, Addis Ababa University, Addis Ababa, Ethiopia which is the only public oncologic care institution in the country currently. Newly diagnosed breast cancer patients who came to the center for breast cancer care during mid-June 2014 to mid-June 2015 were enrolled and monitored for hematologic toxicity and RDI until completion of their chemotherapy cycles. Study participants were followed up for 3–6 months depending on the chemotherapy regimen received and the number of cycles recommended by their respective clinician. All consecutive and volunteer adult female patients, pathologically and clinically diagnosed with breast cancer (stages I–IV) who received neo-adjuvant, adjuvant or palliative chemotherapy regimen in the outpatient day care ward and those with no prior history of anemia were included. These patients were those who took the first cycle of chemotherapy in this radiotherapy center and had been on follow-up of their chemotherapy. Patients who were less than 18 years of age, pregnant, with multiple primary tumor types, patients on concurrent radiotherapy, or patients who started chemotherapy at private clinic were excluded.

The main aim of this study was to determine the incidence and predictors of chemotherapy induced hematologic toxicities and reduced RDI. The sample size required to detect a risk ratio of 2.0 for grade 3 or 4 hematologic toxicity with 95% confidence and 80% power, and assuming 8% loss to follow up is calculated to be 269 (Dean et al., 2013). Ethical clearance was obtained from Institutional Review Board (IRB) of the college of health sciences, Addis Ababa University, Armauer Hansen Research Institute Ethical Review Committee (AAERC), and National Research Ethics Review Committee (NRERC) of the federal democratic republic of Ethiopia. Signed informed consent was obtained from individual patient prior to participation in the study.

### Treatment, Follow Up, and Laboratory Analysis

The common chemotherapy regimens in the outpatient care at Tikur Anbessa Hospital comprised of six cycles of FAC (5-Flourouracil 500 mg/m^2^, Adriamycin [Doxorubicin] 50 mg/m^2^, and Cyclophosphamide 500 mg/m^2^), four cycles of AC (Adriamycin 50 mg/m^2^ and Cyclophosphamide 600 mg/m^2^), six cycles CMF (Cyclophosphamide 500 mg/m^2^, Methotrexate 40 mg/m^2^ and Fluorouracil 500 mg/m^2^, and sequential AC – T (four cycles of Adriamycin 60 mg/m^2^ and Cyclophosphamide 600 mg/m^2^ followed by another 4 cycles of Taxol 175 mg/m^2^).

Cycles were 3 weeks long (planned every 21 days) in the study setup. A dose reduction was described as ratio of the actual dose given compared with the standard or planned dose. Dose delay was described as the actual cycle length compared with standard or planned cycle length. A dose reduction was defined as a ≥15% reduction relative to standard and dose delay was defined as ≥15% delay in days relative to the standard cycle length for chemotherapy (Wildiers and Reiser, 2011).

At baseline, patients’ medical records, biopsy reports, radiologic imaging results [X-RAY, ultrasound, CT scan (where available)] and laboratory investigations (blood counts and organ function estimates [Alkaline phosphatase (ALP), alanine aminotransferase (ALT) and aspartate aminotransferase (AST), serum creatinine (SCr), and blood urea nitrogen (BUN)] levels were recorded. Pretreatment demographic, clinical and tumor characteristics collected include age at diagnosis, menopausal status, performance status, weight, height, body mass index (BMI, classified based on World Health Organization criteria), body-surface area (BSA), site of tumor, histologic type of the tumor, degree of differentiation, tumor size, lymph node involvement, tumor stage at diagnosis, tumor receptor status, co-morbidity (assessed and classified based on the Charlson Co-morbidity Index) (Breccia et al., 2011), chemotherapy panel, i.e., treatment intent (neo-adjuvant, adjuvant, or metastatic), chemotherapy regimen (planned dose and schedule), and primary or secondary G-CSF use. In the subsequent cycles, follow-up clinical information including actual chemotherapy dose received and date of administration, blood laboratory investigations, dose intensity reducing events (i.e., dose reduction and/or delays) occurred across the cycles were also recorded.

### *CYP2B6, CYP2C9, CYP2C19, CYP3A5, CYP2J2, ABCB1*, and *POR* Genotyping

Genomic DNA was isolated from peripheral leukocytes in whole blood samples using QIAamp DNA Midi Kit (Qiagen GmbH, Hilden, Germany). Genotypes of common functional variant alleles of drug metabolizing enzymes’ genes relevant for cyclophosphamide disposition or bioactivation including, *CYP2B^∗^6, CYP2C9^∗^2, CYP2C9^∗^3, CYP2C19^∗^2, CYP2C19^∗^3, CYP3A5^∗^3, CYP2J2^∗^7*, and *POR^∗^28* were carried out using Taqman^^®^^ allele specific PCR (Applied Biosystems Genotyping Assays) as described previously (Hatta and Aklillu, 2015). In brief genotyping was performed using TaqMan^^®^^ drug metabolism genotyping assay reagents for allelic discrimination (Applied Biosystems Genotyping Assays) with the following ID numbers for each SNP: C__7817765_60 for *CYP2B6^∗^6* (c.516G4T, rs3745274), C__26201809_30 for *CYP3A5^∗^3* (c.6986A4G, rs776746), C__25625805_10 for *CYP2C9^∗^2* (rs1799853), C__27104892_10 for *CYP2C9^∗^3* (rs1057910), C__25986767_70 for *CYP2C19^∗^2* (rs4244285), C__27861809_10 for *CYP2C19^∗^3* (rs4986893), C_9581699_80 for *CYP2J2^∗^7* (rs890293), C_8890131_30 for *POR^∗^28* (rs1057868), and C__11711730_20 for *ABCB1* (rs3842). Genotyping was carried out using QuantStudio 12K Flex Real-Time PCR system (Life Technologies Holding, Singapore, Singapore). The final volume for each reaction was 10 μL, consisting of TaqMan^^®^^ fast advanced master mix (Applied Biosystems, Waltham, MA, United States), TaqMan 20X drug metabolism genotyping assays mix (Applied Biosystems) and genomic DNA. The PCR profile consisted of an initial step at 60°C for 30 s, hold stage at 95°C for 10 min and PCR stage for 40 cycles step 1 with 95°C for 15 min and step 2 with 60°C for 1 min and after read stage with 60°C for 30 s.

### Study Outcomes

The primary outcome measure was incidence of grade 3 or 4 hematologic toxicity during the course of chemotherapy. Toxicity events were graded according to the National Cancer Institute Common Terminology Criteria for Adverse Events (CTCAE), version 4.0 (CTCAE, 2010). Accordingly, grades 3 and 4 anemia were defined, respectively, as hemoglobin value of 6.5–8.0 g/dL and <6.5 g/dL. Platelet counts between 25,000 and 50,000/ mm^3^ and <25,000/mm^3^ were classified as grade 3 and 4 thrombocytopenia, respectively. Neutropenia was defined as grade 3 (neutrophil count 500 – < 1,000/ mm^3^) and grade 4 (neutrophil count < 500/mm^3^). Grade 2 toxicities were also considered to be grade 2 anemia (hemoglobin level 8.0–10.0 g/dL), grade 2 thrombocytopenia (platelets count between 50,000 and 5,000/mm^3^), grade 2 neutropenia (neutrophil count 1000 – < 1,500/mm^3^).

The secondary outcome was the average received RDI, defined as the proportion of the reference standard dose-intensity for each regimen actually received. Then, the proportion of patients receiving less than 85% of the reference dose was determined. Both planned and unplanned reductions in RDI (<85%) were calculated for each chemotherapy panels (neo-adjuvant, adjuvant and metastatic). The dose intensity (DI) of each agent was calculated by dividing the total received dose of the agent to the total number of weeks of treatment. The RDI of each agent was expressed as the total delivered dose of the agent per unit time (i.e., weeks) as a percentage of the standard/planned dose. The RDI for each regimen represents the average RDI for each chemotherapeutic agent in a given regimen (Hryniuk et al., 1998). The standard reference dose intensity for each drug was considered to be the established dose in clinical trials in mg/m^2^ per unit time (in weeks) (Mamounas et al., 2005).

### Statistical Analysis

Descriptive statistics was used to explore the demographic characteristics and clinical profiles including, toxicities, RDI, dose delays, and dose reductions of participants. Categorical variables were reported as percentages. Confidence intervals (95%) were computed for incidence proportion of overall grade 3 or 4 hematologic toxicities. Continuous variables were diagnosed for normality of the distribution and presented as mean ± standard deviations (SD) or median with inter quartile range (IQR). Paired sample *t*-test was used to compare the mean values of absolute neutrophil counts across the cycles and to determine if there were significant changes in neutropenic toxicities at each cycle compared to baseline.

Chi Square (χ^2^) test was used to evaluate the allele and genotype frequencies if the patient population is in Hardy-Weinberg equilibrium (HWE). Cox proportional hazard regression model was used to estimate hazard risks factors for grade 3 or 4 hematologic toxicities. Results were expressed as hazard ratios (HRs) and 95% confidence intervals. Logistic regression analysis was performed to identify predictors of reduced RDI and the results were expressed as Odds ratios (OR) and 95% confidence intervals (CI). RDI categorization was based on the threshold of RDI < 85% and RDI ≥ 85%. In all multivariate regression models, variables with *p* ≤ 0.20 in univariate analysis were used. Hosmer–Lemeshow goodness of fit test was used to assess the model fit (Hosmer-Lemeshow statistic ≥ 0.05). Backward elimination (likelihood ratio) was used as the variable selection method. The data were analyzed using SPSS for windows, version 21.0. A *p* < 0.05 was considered statistically significant for each test and then *Bonferroni* correction (as the number of hypotheses is fairly small) was applied for multiple comparisons.

## Results

### Socio-Demographic Characteristics

A total of 285 patients were enrolled and followed up in the study. Of these, 36 patients were excluded: six patients had taken previous chemotherapy for breast cancer or non-Hodgkins lymphoma (NHL), 5 patients received concurrent radiotherapy, 6 patients were pregnant, 4 patients started chemotherapy at a private clinic and were referred to the radiotherapy center of the hospital (complete information was unavailable), 11 patients were lost to follow up for the second and subsequent cycles, 4 patients had poor performance (Karnosfsky’s performance scale < 60). Thus, data from a total of 249 breast cancer patients were followed up and included in the analysis. The socio-demographic profiles of the patients are given in Table 1 below. Larger proportions (73.5%) of patients were 45 years of age or younger (median age 39 years). Six (2.4%) and 31.3% patients had BSA range ≥ 2 and BMI range ≥ 25 kg/m^2^, respectively. Twenty four (9.6%) and 11 (4.4%) patients were co-morbid with cardiovascular diseases and HIV/AIDS, respectively. Forty nine (19.7%) and six (2.4%) patients initiated their chemotherapy at baseline absolute neutrophil count of 1,500–2,500/m^2^ (grade 1 neutropenia) and 1,000–1,500 m^2^ (grade 2 neutropenia), respectively.

**Table 1:** Socio-demographic characteristics of breast cancer patients at the radiotherapy center, Tikur Anbessa specialized hospital, Addis Ababa University, Ethiopia, 2015.

Parameters	Statistic
Age (years) (median + IQR)^∗^	39 + 14
BSA (m^2^) (mean ± SD)	1.6 ± 0.17
BMI (Kg/m^2^) (mean ± SD)	23.9 ± 4.65
Presence of co-morbidities, n (%)	40 (16.1%)
Cardiovascular co-morbidities, n (%)	24 (9.6%)
Presence of HIV/AIDS, n (%)	11 (4.4%)
Presence of diabetes, n (%)	3 (1.2%)
**Baseline laboratory results**	
WBC (10^3^/mm^3^) (median + IQR)	6.60 + 2.6
ANC (10^3^/mm^3^) (median + IQR)	3.48 + 1.98
Hgb (gm/dL) (median + IQR)	13.8 + 1.67
HCT (%) (mean ± SD)	41.3 ± 3.62
PLT (10^3^/mm^3^) (median + IQR)	291 + 108
ALT (U/L) (median + IQR)	20 + 14
AST (U/L) (median + IQR)	25 + 12.25
ALP (U/L) (median + IQR)	241 + 163
SCr (mg/dL) (mean ± SD)	0.87 ± 0.18
BUN (mg/dL) (median + IQR)	18 + 12

*^∗^ALP, Alkaline phosphatase; ALT, alanine aminotransferase; ANC, absolute neutrophil count; AST, aspartate aminotransferase; BUN, Blood urea nitrogen; BSA, body surface area; Hgb, hemoglobin; HCT, hematocrit; IQR, inter quartile range; PLT, platelet count; SCr, Serum creatinine; SD, standard deviation; WBC, white blood cell count.*

In this cohort of patients, invasive ductal carcinoma was the most common (85.9%) type of cancer and larger proportion (73.1%) of patients had node positive tumor (Table 2). Fifty five (22%) patients had at least two secondary involved organs that included to the lung (37 patients) or liver (28 patients). Hormone receptor status was obtained from 24 patients and triple negative breast cancer accounted for 25%. Adjuvant chemotherapy was given to 71.5% of the patients. Majority (65.9%) of the patients in all chemotherapy panels (neo-adjuvant, adjuvant or metastatic) received 5-Flourouracil, Doxorubicin, and Cyclophosphamide (FAC). The mean doses of these drugs administered per cycle were 797.5 mg ± 77.37 (5-Flourouracil) and 822.08 mg ± 124.3 (Cyclophosphamide), while the median Doxorubicin dose administered was 82.5 mg (IQR 10.0). None of the study participants received primary colony stimulating factor (G-CSF), however, 4.8% of the patients received it during the course of chemotherapy in the subsequent cycles.

**Table 2:** Tumor characteristics of breast cancer patients, at the radiotherapy center, Tikur Anbessa specialized hospital, Addis Ababa University, Ethiopia, 2015.

Parameters		Frequency, n (%)
Histologic type of invasive cancer	Ductal	214 (85.9%)
	Lobular	18 (7.2%)
	Mixed type	7 (2.8%)
	Special type	9 (3.6%)
Histologic grade	Grade I	26 (17.1%)
	Grade II	79 (52%)
	Grade III	47 (30.9%)
Positive nodal involvement		182 (73.1%)
Hormone receptor and Her2 expression	ER +	15 (62.5%)
	PR +	12 (50%)
	HER-2/neu	6 (25%)
	Triple negative	6 (25%)
Distant metastatic site	No known metastasis	52 (21.3%)
	Bone, lymph node, or lung only	140 (56.2%)
	Liver, CNS, Lung + other organs)	55 (22.1%)
Chemotherapy setting	Neoadjuvant	16 (6.4%)
	Adjuvant	178 (71.5%)
	Palliative	55 (22.1%)
Chemotherapy regimen	^♣^FAC	164 (65.9%)
	AC	48 (19.3%)
	AC – T	34 (13.7%)
	CMF	3 (1.2%)

*^♣^FAC (5-Flourouracil 500 mg/m^*2*^, Adriamycin [Doxorubicin] 50 mg/m^*2*^, and Cyclophosphamide 500 mg/m^*2*^); AC (Adriamycin 50 mg/m^*2*^ and Cyclophosphamide 600 mg/m^*2*^); CMF (Cyclophosphamide 500 mg/m^*2*^, Methotrexate 40 mg/m^*2*^, and Fluorouracil 500 mg/m^*2*^; AC – T (Adriamycin 60 mg/m^*2*^ and Cyclophosphamide 600 mg/m^*2*^ followed by Taxol 175 mg/m^*2*^).*

### Incidence and Predictors of Chemotherapy Induced Grade 3 or 4 Hematological Toxicity

The overall incidence of chemotherapy induced grade 3 or 4 hematological toxicity was 51.0% [95% confidence interval (CI) = 44.54–57.46%]. Most of the hematologic toxicity events were neutropenic toxicities, 50.2% (95% CI = 43.83–56.56%). The incidence of grade 3 or 4 anemia and thrombocytopenia across the cycles was 2 and 1.2%, respectively. Paired sample *t-*test showed there was significant decrease in the mean absolute neutrophil counts in each cycles compared to the baseline (*p* < 0.05 for the trend across the cycles).

**FIGURE 1:**
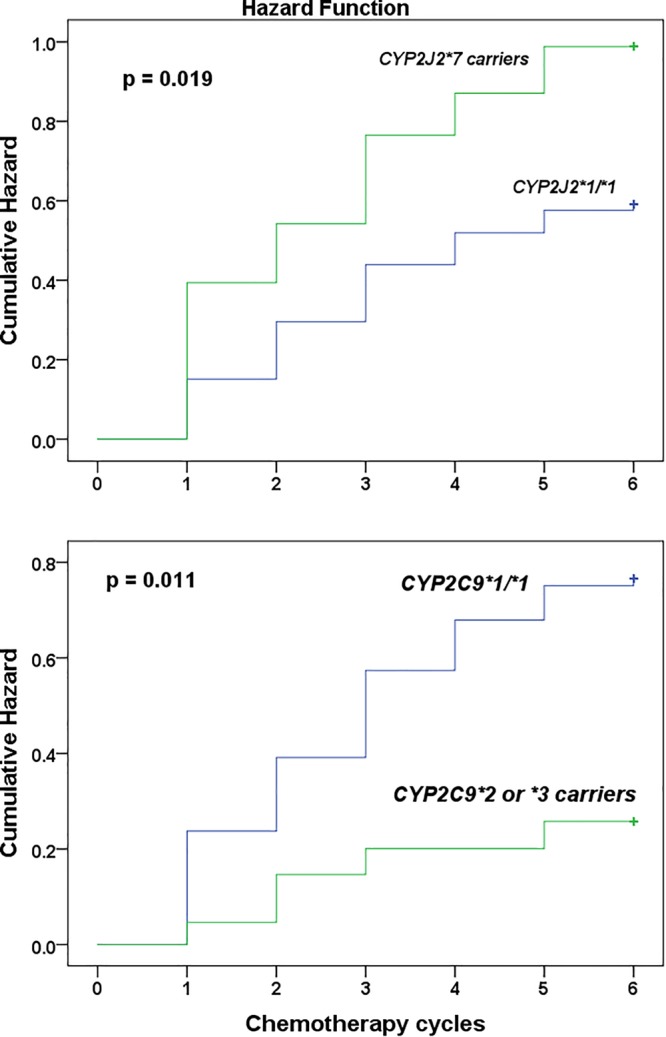
Kaplan–Meier curves to estimate cumulative hazard for the development of chemotherapy induced grade 3 hematologic toxicity stratified by *CYP2J2*^∗^7 (top) and *CYP2C9* (below) genotype among Ethiopian female breast cancer patients at the radiotherapy center, Tikur Anbessa specialized hospital, Addis Ababa University, Ethiopia, 2015.

The overall allele frequencies of *CYP2B6^∗^6, CYP2C9^∗^2, CYP2C9*^∗^*3, CYP2C19^∗^2, CYP2C19^∗^3, CYP3A5^∗^3, CYP2J2^∗^7, POR^∗^28*, and *ABCB1 rs3842G* variant alleles were 34.8, 5.8, 1.5, 15.9, 1.8, 62.5, 15.2, 15.5, and 13.2%, respectively. All genotype frequencies were in consistent with HWE (*p* > 0.05). Comparison of genotype and allele frequencies between patients who developed chemotherapy induced grade 3 or 4 hematological toxicity versus treatment tolerant is presented in Table 3. After multiple comparisons, patients carrying *CYP2C9* ^∗^2 or ^∗^3 alleles had significantly lower incidence of hematologic toxicity (3.1% versus 11.4%, *p* = 0.024) (Table 3). Controlling for *POR* genotype, grade 3 or 4 hematologic toxicity was significantly lower in patients with *CYP2C9* ^∗^2 or ^∗^3 alleles who also carried *POR*^∗^28 (*p* = 0.003). No such association was observed among *POR*^∗^1/^∗^1 carriers (*p* = 0.12). No interaction was also detected between POR and other genotypes (*p* > 0.05). On the other hand, the cumulative Kaplan–Meier hazard curves (Figure 1) showed association for the development hematologic toxicities among *CYP2J2* and *CYP2C9* genotype. There was no significant association between *CYP2B6, CYP2C19*, or *POR^∗^28* and *ABCB1* genotype with risk for hematologic toxicity.

**Table 3:** Genotype and allele frequencies of candidate drug metabolizing enzymes genes by hematologic toxicities, at the radiotherapy center, Tikur Anbessa specialized hospital, Addis Ababa University, Ethiopia.

			Grade 3 or 4	
			hematologic	
	Gene	Genotype	toxicity		*p*-value
			**No**	**Yes**	
			**n (%)**	**n (%)**	

**Genotype**	*CYP2B6*	^∗^1/^∗^1	34 (40.9)	35 (43.2)	0.51
**frequency**		^∗^1/^∗^6	37 (44.6)	39 (48.1)	
		^∗^6/^∗^6	12 (14.5)	7 (8.6)	
	*CYP2C9*	^∗^1/^∗^1	66 (79.5)	76 (93.8)	0.021
		^∗^1/^∗^2 or ^∗^3	15 (18.1)	5 (6.2)	
		^∗^2/^∗^2	2 (2.4)	0 (0)	
	*CYP2C19*	^∗^1/^∗^1	57 (68.7)	55 (67.9)	0.68
		^∗^1/^∗^2 or ^∗^1/^∗^3	24 (28.9)	22 (4.9)	
		^∗^2/^∗^2 or ^∗^3/^∗^3	2 (2.4)	4 (4.9)	
	*CYP3A5*	^∗^1/^∗^1	17 (20.5)	6 (7.4)	0.052
		^∗^1/^∗^3	37 (44.6)	40 (49.4)	
		^∗^3/^∗^3	29 (34.9)	35 (43.2)	
	*POR*	^∗^1/^∗^1	56 (67.5)	60 (74.1)	0.09
		^∗^1/^∗^28	27 (32.5)	18 (22.2)	
		^∗^28/^∗^28	0 (0)	3 (3.7)	
	*CYP2J2*	^∗^1/^∗^1	67 (80.7)	54 (66.7)	0.12
		^∗^1/^∗^7	13 (15.7)	23 (28.4)	
		^∗^7/^∗^7	3 (3.6)	4 (4.94)	
		Allele			
**Allele**	*CYP2B6*	^∗^6	61 (36.7)	53 (32.7)	0.44
**frequency**
	*CYP2C9*	*^∗^2*	15 (9.04)	4 (2.5)	0.011
		*^∗^3*	4 (2.4)	1 (0.6)	0.19
		^∗^2 or ^∗^3	19 (11.4)	5 (3.1)	0.004
	*CYP2C19*	*^∗^2*	26 (15.7)	26 (16.1)	0.92
		*^∗^3*	2 (1.2)	4 (2.5)	0.39
		^∗^2 or ^∗^3	28 (16.9)	30 (18.5)	0.7
	*CYP3A5*	^∗^3	95 (57.2)	110 (67.9)	0.046
	*POR*	^∗^28	27 (16.3)	24 (14.8)	0.72
	*CYP2J2*	^∗^7	19 (11.4)	31 (19.1)	0.041

Univariate followed by multivariate Cox proportional hazard regression analysis were done to model predictors of grade 3 or 4 hematologic toxicities (Table 4). To avoid the interaction between pretreatment white blood cells count (WBC) and absolute neutrophils count (ANC) in the regression model (as neutrophils account for about 45–70% of WBC), two multivariate regression models were developed. First, all variables selected from univariate analysis, excluding ANC, were entered into multivariate model. In model 2, all variables except WBC were modeled. The result indicated that *CYP2J2^∗^7* allele (HR = 1.819; 95% CI, 1.141–2.899, *p* = 0.012) and low baseline WBC count (grade 1 leukocytopenia) (HR = 2.748; 95% CI, 1.466–5.149, *p* < 0.001) were independent predictors of toxicity (model 1, Table 4). In model 2, low baseline ANC count (grade 1 or 2 neutropenia) (HR = 2.746; 95% CI, 1.732–4.353, *p* < 0.001), and *CYP2J2^∗^7* allele (HR = 1.735; 95% CI, 1.085–2.775, *p* = 0.021) (model 2) were strong independent risk factors of hematologic toxicity. Thus, patients who took chemotherapy with low baseline white blood cells count, or low neutrophils count were at increased risk of experiencing grade 3 or 4 hematologic toxicity in the subsequent cycles. In addition, the risk of toxicity was also higher in patients carrying *CYP2J2^∗^7* variant allele. Although Chi square test showed association of *CYP2C9* variant alleles to toxicity (Table 3), these alleles did not reach to statistical significance in the final multivariate regression analysis (Table 4).

**Table 4:** Cox proportional hazard regression results for incidence of grade 3 or 4 hematologic toxicity.

Parameters	Univariate analysis		Multivariate analysis			
	^†^OR (95% CI)	*p*-value	Model 1		Model 2	
			OR (95% CI)	*p*-value	OR (95% CI)	*p*-value
**Body surface area**		
<1.5	1					
1.5–1.75	0.68 (0.45–1.04)	0.07				
>1.75	1.03 (0.62–1.72)	0.91				
**Cardiovascular co-morbidity**		
No	1					
Yes	0.56 (0.27–1.14)	0.11				
**HIV/AIDS**		
No	1					
Yes	0.62 (0.30–1.26)	0.19				
**Baseline leukocyte toxicity grade**		
Normal^‡^	1		1			
Grade 1^£^	3.20 (2.02–5.08)	< 0.001	2.75 (1.47–5.15)	< 0.001		
**Baseline neutrophil toxicity grade**		
Normal‡	1				1	
Grade 1^§^ or Grade 2^¥^	2.32 (1.58–3.41)	< 0.001			2.75 (1.73–4.35)	< 0.001
***CYP3A5***		
^∗^1/^∗^1	1		1		1	
^∗^3 carriers	2.34 (1.03–5.32)	0.042	2.04 (0.87–4.79)	0.05	2.12 (0.90–4.96)	0.08
***CYP2C9***		
^∗^1/^∗^1	1		1		1	
^∗^2 or ^∗^3 carriers	0.35 (0.14–0.88)	0.024	0.40 (0.13–1.18)	0.10	0.49 (0.19–1.22)	0.12
***CYP2C19***		
^∗^1/^∗^1	1					
^∗^2 or ^∗^3 carriers	1.08 (0.76–1.54)	0.66				
***CYP2J2***		
^∗^1/^∗^1	1		1		1	
^∗^7 carriers	1.66 (1.05–2.64)	0.032	1.82 (1.14–2.9)	0.012	1.74 (1.09–2.78)	0.021
***POR***		
^∗^1/^∗^1	1					
^∗^28 carriers	0.95 (0.58–1.56)	0.83				
***ABCB1 rs 3842 A>G***		
AA	1					
G carriers	0.75 (0.35–1.62)	0.46				

*^†^Odds ratio; ^‡^4,500–10,000/m^*3*^; ^£^3,000–4,500/mm^*3*^; ^d'^2,500–7,500/m^*3*^; ^§^1,500–2,500/m^*3*^; ^¥^1,000–1,500/m^*3*^*.

### Incidence and Predictors of Relative Dose Intensity

The overall actual average RDI was 81.9% (95% CI = 80.52–83.28%). Dose delay > 15% of the planned days was observed in 61.4% patients, whereas dose reduction was observed in 11.2% patients. The proportion of patients who received reduced RDI < 85% of the standard/planned dose intensity was 56.6% (95% CI = 50.32–62.88%). Three point six percent of the reduced RDI was planned dose reduction from the start of therapy as decided by senior oncologists to reduce the dose in metastatic conditions or due to co-morbidities. The remaining was unplanned RDI reduction associated with subsequent dose/treatment delays due to toxicities.

Results of multivariate logistic regression analysis (Table 5) showed that the independent risk predictors of reduced RDI were BMI ≤ 18.4 kg/m^2^ (underweight) (AOR 5.975; 95% CI = 1.361–26.227, *p* = 0.018), low baseline leukocyte count (AOR 6.092; 95% CI = 1.238–29.98, *p* = 0.026), low baseline neutrophils count (AOR 3.37; 95% CI = 1.412–8.045, *p* = 0.006) and *CYP2J2^∗^7* allele (AOR 2.892; 95% CI = 1.238–6.647, *p* = 0.012). On the other hand, the odds of receiving RDI less than 85% of the dose intensity was significantly lower in patients with *CYP2B6 ^∗^6/^∗^6* genotype (AOR 0.179; 95% CI = 0.049–0.656, *p* = 0.009) irrespective of other factors.

**Table 5:** Logistic regression results for predictors of reduced RDI.

Parameters	Univariate analysis		Multivariate analysis			
	OR^†^ (95% CI)	*p*-value	Model 1	*p*-value	Model 2	*p*-value
			OR (95% CI)		OR (95% CI)	
**BSA (m^2^)**		
< 1.5	1.57 (0.82–3.02)	0.17	–		–	
1.5–1.75	1		–		–	
>1.75	0.81 (0.41–1.59)	0.53	–		–	
**BMI (Kg/m^2^)**		
≤18.4	2.34 (0.88–6.24)	0.09	5.98 (1.36–26.2)	0.02	6.50 (1.45–29.05)	0.01
18.5–24.9	1		1		1	
25–29.9	0.92 (0.48–1.75)	0.79	0.75 (0.32–1.79)	0.52	0.79 (0.33–1.88)	0.59
≥30	0.68 (0.30–2.52)	0.35	0.65 (0.22–1.94)	0.44	0.57 (0.18–1.75)	0.32
**Chemotherapy regimen**		
FAC	1		–	–	–	–
CMF	0.41 (0.04–4.62)	0.47	–	–	–	–
AC	2.21 (1.09–4.49)	0.03	–	–	–	–
AC-T	0.65 (0.31–1.37)	0.26	–	–	–	–
**Cardiovascular co-morbidity**		
No	1		–		–	
Yes	0.42 (0.18–1.01)	0.05	–	–	–	–
**Baseline leukocyte toxicity**		
Normal^‡^	1		1		–	–
Grade 1^£^	6.52 (1.89–22.41)	0.003	6.09 (1.24–29.98)	0.026	–	–
**Baseline neutropenic toxicity**		
Normal‡	1		–		1	
Grade 1^§^ or 2^¥^	2.78 (1.42–5.43)	0.003	–		3.37 (1.41–8.05)	0.006
***CYP2B6***		
^∗^1/^∗^1	1		1		1	
^∗^1/ ^∗^6	0.96 (0.52–1.79)	0.9	1.47 (0.73–2.99)	0.29	1.63 (0.79–3.36)	0.19
^∗^6/^∗^6			0.18 (0.05–0.66)	0.01	0.19 (0.06–0.77)	0.01
***CYP2C9***		
^∗^1/^∗^1	1		–		–	–
^∗^2 or ^∗^3 carriers	0.87 (0.35–2.13)	0.17	–		–	–
***CYP3A5***		
^∗^1/^∗^1	1		–		–	–
^∗^3 carriers	1.79 (0.075–4.26)	0.17	–		–	–
***CYP2J2***		
^∗^1/^∗^1	1		1		1	
^∗^7 carriers	1.72 (0.84–03.50)	0.14	2.89 (1.26–6.65)	0.012	2.79 (1.21–6.46)	0.02
**ABCB1**		
AA	1	0.83				
Carriers of G	0.92 (0.43–1.97)					

*^†^Odds ratio; ^‡^4,500–10,000/m^*3*^; ^£^3,000–4,500/mm^*3*^; ^d'^2,500–7,500/m^*3*^; ^§^1,500–2,500/m^*3*^; ^¥^1,000–1,500/m^*3*^.*

## Discussion

In the present study, we prospectively investigated the incidence of chemotherapy induced hematologic toxicity, the proportion of reduced RDI, and associated risk factors including pharmacogenetics in drug mobilizing enzymes relevant for the disposition or bio-activation of chemotherapeutic agents. Our main finding include (i) higher incidence of grade 3 or 4 hematologic toxicities (51%), which was largely manifested as neutropenic toxicity (50.2%), that caused treatment delay in large number of patients. Consequently, significant proportion of the patients (56.6%) received reduced RDI; (ii) Low baseline WBC and neutrophil count is a strong predictor of both chemotherapy induced hematologic toxicity and reduced RDI; (iii) significant association of *CYP2J2^∗^7* and *CYP2C9* genotype with chemotherapy induced hematologic toxicity, and *CYP2B6* genotype with reduced RDI. To the best of our knowledge, this is the first study to investigate the incidence and predictors of anticancer chemotherapy induced hematological toxicities, reduced chemotherapy dose intensities and associated risk factors among Ethiopian breast cancer patients, and the first pharmacogenetics association study for chemotherapy induced hematologic toxicity and RDI in Sub-Saharan Africa population.

Cyclophosphamide, the cornerstone of breast cancer chemotherapy in Ethiopia, is mainly metabolized to 4-hydroxy-cyclophosphamide by genetically polymorphic *CYP3A, CYP2B6, CYP2C9, CYP2C19*, and *CYP2J2* enzymes (El-Serafi et al., 2015). We investigated the association of common functional genetic variant alleles of these enzymes with chemotherapy induced hematologic toxicity and RDI. We found that, patients with *CYP2J2^∗^7* allele, baseline grade 1 leukocytopenia, and grade 1 or 2 neutropenia, were significantly associated with increased risk of grade 3 or 4 hematologic toxicity. Low baseline WBC count as an important predictor for chemotherapy induced hematologic toxicity was consistent with previous reports (Lyman et al., 2003; Jenkins et al., 2012; Tsuji et al., 2016). However, *CYP2J2^∗^7* was identified as new pharmacogenetic risk factors of chemotherapy induced hematologic toxicity in Ethiopian breast cancer patients. *CYP2J2* is an epoxygenase enzyme that catalyzes the metabolism of structurally diverse therapeutic compounds, particularly in extra-hepatic tissues (Berlin et al., 2011). A recent study reported the role of *CYP2J2* in the bioactivation of cyclophosphamide (El-Serafi et al., 2015). Using enzyme kinetic studies, the authors revealed that *CYP2J2* is over expressed during cyclophosphamide treatment, and the bioactivation of the drug was significantly correlated to *CYP2J2* expression. In another study, *CYP2J2* is reported to be highly expressed in human and mouse hematological cell lines, as well as in peripheral blood and bone marrow cells of leukemia patients (Chen et al., 2011). The cytotoxic effect of cyclophosphamide in hematological cell lines has also been associated with *CYP2J2* expression, despite the lack of *CYP2B6* (Xie et al., 2002). Interestingly, we found increased risk for grade 3 or 4 chemotherapy induced hematologic toxicities in carriers of *CYP2J2^∗^7*. As *CYP2J2^∗^7* is associated with increased enzyme activity (El-Serafi et al., 2015), and patients carrying this variant allele could possibly activate cytotoxic agents such as cyclophosphamide to 4-hydroxycyclophosphamide at a faster rate and hence increased risk for toxicity.

The frequency distribution of *CYP2C9* genotype and defective variants alleles were significantly different between patients who developed grade 3 or 4 chemotherapy induced hematologic toxicities versus treatment tolerant (Table 3). *CYP2C9^∗^2* or *^∗^3* allele frequency was significantly higher in treatment tolerant (11.4%) than chemotherapy induced hematologic toxicities cases (3.1%) or the general random Ethiopian population. The frequency of *CYP2C9^∗^2* and *CYP2C9^∗^3* alleles in healthy Ethiopians is 4 and 2%, respectively. Furthermore, univariate regression analysis indicated a significant association of *CYP2C9* with grade 3 or 4 chemotherapy induced hematologic toxicities. Our result indicates that being carriers of defective variant alleles *CYP2C9^∗^2* or ^∗^3 alleles and hence reduced *CYP2C9* enzyme activity is associated with a lower risk for chemotherapy induced hematologic toxicities. This is in line with a previous study reporting a threefold lower intrinsic clearance of cyclophosphamide CYP2C9.2 and CYP2C9.3 compared to CYP2C9.1 (Griskevicius et al., 2003). Thus, reduced cyclophosphamide bioactivation, in carriers of defective *CYP2C9* variant alleles, may be protective against cyclophosphamide induced hematologic toxicities. On the other hand, reduced cyclophosphamide bioactivation, in patients carrying *CYP2C9* defective alleles, may compromise the treatment success, increase risk of recurrence as well as worsen survival outcome. Consequently, future studies evaluating treatment successes based on *CYP2C9* genotype is needed to identify whether genotype-based cyclophosphamide dose modification is required or not. Although *CYP2C9* genotype was retained in the final multivariate regression model, the *p*-value did not reach significance, which might be due to a lower variant allele frequency and hence sample size.

Pharmacologically, the cytotoxic properties of chemotherapeutic agents form the basis for their anticancer as well as myelosuppressive effects (Kozma et al., 2012). In early stage breast cancer patients receiving CMF, greater myelosuppression during treatment had been shown to have better outcomes (Mayers et al., 2001). This indicates that chemotherapy induced hematologic toxicities could be predictive of anticancer response. In clinical practice, the primary response to hematologic toxicity during anticancer chemotherapy is dose reduction and/or treatment delays to allow cells to regenerate (Piccart et al., 2000). While toxicity could be avoided or reduced by varying schemes of dose attenuation and treatment delay, it is done at the expense of reduction in the dose intensity received.

Reductions of dose intensity from standard dose and dose-intensity of CMF may compromise desired survival advantages in breast cancer patients (Bonadonna et al., 1995; Piccart et al., 2000). Superior efficacy and survival benefits (both disease-free survival and overall survival) have been observed in those patients who received greater dose-intensity of CAF (Budman et al., 1998). The key role of dose density and intensity has also been substantiated with a study that compared dose-dense (14 days cycles with primary prophylactic GCSF support) and standard (21 days cycles) schedules in patients with node-positive breast cancer. Dose-dense schedules are associated with significantly improved disease-free and overall survival (Citron et al., 2003). In the subsequent studies, higher RDI has been shown to have the greatest impact particularly for patients with early-stage disease in which curative intent with adjuvant chemotherapy is the goal (Sandy and Della-Fiorentina, 2013; Vavra et al., 2013; Havrilesky et al., 2015).

RDI of less than 85% is widely considered as a benchmark for clinically important reduction in chemotherapy dose-intensity (Bonadonna et al., 2005; Wildiers and Reiser, 2011). Although achieving this RDI level is important to gain improved outcome, many patients in various clinical settings are treated with a lower dose intensity of chemotherapy (Piccart et al., 2000; Sandy and Della-Fiorentina, 2013). The present study result estimates that, the prevalence of reduced RDI received (56.6%) among Ethiopian patients could be as low as 50.3% and as high as 62.9%. This indicates that a higher proportion of patients are receiving reduced RDI in our setup unacceptably, compared to the finding reported in Canada (4.4%) (Raza et al., 2009), United States (30%) (Shayne et al., 2006, Shayne et al., 2007), and Australia (12% in adjuvant and 36% metastatic setups) (Bae et al., 2014). In line with the finding of our study, a nationwide survey involving breast cancer patients across the United States who received a similar chemotherapy regimens as our study, reported that 55% of women received less than 85% of the RDI (Lyman et al., 2003). The authors reported dose reductions in 40% of patients, and treatment delays up to 7 days in 24% of patients (Lyman et al., 2003). However, the major RDI reducing event in our study was treatment delay observed in 61.4% patients.

Several risk factors for reduced RDI have previously been reported in the literature including older age, and lower pretreatment blood cell counts, BSA > 2 m^2^, and nonuse of G-CSF (Lyman et al., 2003). The present study has revealed that, underweight (BMI ≤ 18.4 kg/m^2^), baseline grade 1 leukocyte toxicity and grade 1or 2 neutropenia were independent predictors of reduced RDI. Consequently, such patients are more likely to receive reduced RDI. On the other hand, *CYP2B6^∗^6/^∗^6* genotype was associated with a lower risk of receiving reduced RDI. In line with our finding, greater incidence of dose delay was observed during AC treatment in variant carriers of *CYP2B6^∗^2* and *CYP2B6^∗^5* (Bray et al., 2010). Other patient-related factors such as appointment cancelations, patient non-compliance and patient knowledge deficits may also contribute to clinically important reduction in chemotherapy dose-intensity (Piccart et al., 2000; Wildiers and Reiser, 2011; Kozma et al., 2012). *CYP2B6* is the main enzyme that substantially metabolizes cyclophosphamide into its active metabolite (4-hydroxy-cyclopsphmaide), which is known to induce hematologic toxicities. Although *CYP2B6^∗^6* genotype was not identified as a risk factor for hematologic toxicity, we found significant protective effect against reduced RDI. *CYP2B6^∗^6* allele is associated with reduced enzymatic activity and higher incidence of antiretroviral induced liver toxicity in Ethiopian HIV patients (Yimer et al., 2012). Reports regarding the impact of *CYP2B6^∗^6* on chemotherapy induced hematologic toxicity are inconsistent. A recent finding indicated that, being non-*CYP2B6^∗^6* carrier was a significant predictor of grade 4 neutropenia in breast cancer patients treated with doxorubicin and cyclophosphamide combination chemotherapy (Tsuji et al., 2016). By contrast, few other studies concluded that *CYP2B6* genotype was not significantly associated with myelotoxicity (Yao et al., 2010; Haroun et al., 2015). Such inconsistencies could be attributed to the difference in the sample size and study population. Moreover, the relative expression levels of *CYP2J2* and *CYP2B6* may determine *in vivo* kinetics and toxicity of drugs metabolized by these enzymes.

Although this study provides the first data on factors associated with chemotherapy associated hematologic toxicities and RDI, our study may not be powered enough to show significant association of other baseline clinical parameters including co-morbidity status, renal function (serum creatinine and blood urea nitrogen) and liver function estimates (Alkaline phosphatase, alanine aminotransferase, and aspartate aminotransferase) with either hematologic toxicity or reduced RDI. We did not incorporate information concerning potential influence of chemotherapy induced non-hematologic toxicities (such as peripheral neuropathy, fatigue, cardio-toxicity, mucositis, etc.), and patient preference (absenteeism on schedule) on reduced dose intensity. Some of the unplanned reductions in dose-intensity may relate to these factors. It is likely that, differences in PK parameters may further explain the incidence of hematologic toxicity in this cohort of participants. Future larger sample size studies are required to investigate the association of important clinical parameters with hematologic toxicity or reduced RDI in breast cancer.

Studies have shown that prophylactic use of primary G-CSF has been found to be a statistically significant predictor of reduced neutropenic events and thus can reduce the risk of myelosuppresive complications during chemotherapy and help facilitate the delivery of adequate RDI (Citron et al., 2003; Citron, 2004; Lyman et al., 2013; Weycker et al., 2014). However, none of the patients received primary colony stimulating factors in our study. Although the cost of CSF may impede the use of this agent in resource limited countries like Ethiopia, it could be used more cost-effectively if treatment is targeted to patients with identifiable risk factors for subsequent neutropenic complications (Crawford, 2006).

## Conclusion

In conclusion, we report high rates of chemotherapy-induced hematological toxicities causing inadequate RDI in large proportion of Ethiopian breast cancer patients. This study has identified a high activity *CYP2J2^∗^7* and baseline grade 1 or 2 neutropenia is associated with higher risk of chemotherapy induced hematologic toxicity. On the other hand defective *CYP2C9* defective variant alleles and hence low CYP2C9 enzyme activity is protective against developing hematologic toxicity. *CYP2B6^∗^6* genotype, BMI, baseline WBC and neutrophil counts, are predictors of reduced RDI. In general, results from this study provide relevant information to identify patients at greater risk of chemotherapy induced hematologic toxicity and to develop breast cancer treatment guideline in Ethiopia for proper management and patient care. Patients with *CYP2J2^∗^7* alleles and low pretreatment WBC and ANC need prior support such as use the prophylactic use of primary G-CSF before initiation of chemotherapy, close monitoring and treatment follow up accordingly. The potential impact of the observed reduced RDI on the survival outcome in this study population needs further investigation.

## Ethics Statement

Ethical clearance was obtained from Institutional Review Board (IRB) of the college of health sciences, Addis Ababa University, Armauer Hansen Research Institute ethical review committee, and National Research Ethics Review Committee (NRERC) of the federal democratic republic of Ethiopia. Signed informed consent was obtained from individual patient prior to participation in the study.

## Author Contributions

JA, EM, and EA designed the study. JA collected the data. JA and EA did genotyping, analyzed the data and wrote the manuscript. JA, EA, EM, GY, DS, AB, MA, AA, RH, AF, and MH involved in the discussion of results and critical review of the manuscript. All the authors have read and approved the final manuscript.

## Conflict of Interest Statement

The authors declare that the research was conducted in the absence of any commercial or financial relationships that could be construed as a potential conflict of interest.

## Abbreviations

ALP: Alkaline phosphatase
ALT: alanine aminotransferase
ANC: Absolute neutrophil count
AOR: adjusted odds ratio
AST: aspartate aminotransferase
BMI: body mass index
BSA: body-surface area
BUN: blood urea nitrogen
CI: confidence intervals
COR: Crude odds ratio
DI: dose intensity
DFS: disease free survival
G-CSF: granulocyte colony stimulating factor
HWE: Hardy-Weinberg equilibrium
HIV: human immunodeficiency virus
HR: hazard ratio
IRB: Institutional Review Board
MFS: Metastasis free survival
OS: overall survival
QOL: quality of life
RDI: relative dose intensity
SCr: serum creatinine
WBC: white blood cells
